# [Corrigendum] Upregulation of LINC00659 expression predicts a poor prognosis and promotes migration and invasion of gastric cancer cells

**DOI:** 10.3892/ol.2023.13785

**Published:** 2023-04-03

**Authors:** Pihai Gong, Ying Xu, Min Liu, Xiaohui Shen, Yuhang Mao, Yiping Li, Kun Zhang, Shenling Yu, Hong Fan

Oncol Lett 22: 557, 2021; DOI: 10.3892/ol.2021.12818

Following the publication of the above article, a concerned reader drew to the authors’ attention that, for the cell migration and invasion assay experiments shown for the AGS cell line in Fig. 4C on p. 6, the data shown for the ‘Migration / sh-NC’ and ‘Invasion / sh-LINC00659-1’ panels were overlapping, such that these data appeared to have been derived from the same original source, even though they were intended to show the results from differently performed experiments.

The authors have examined their original data, and realize that the data were inadvertently selected incorrectly for the ‘Migration / sh-NC’ panel. Consequently, the corrected version of Fig. 4, featuring the correct data for the ‘Migration / sh-NC’ experiment with the AGS cell line in Fig. 4C, is shown on the next page. The overall conclusions of this study were not affected by this error. All the authors agree to the publication of this corrigendum, and are grateful to the Editor of *Oncology Letters* for allowing them the opportunity to publish this; furthermore, they apologize to the readership for any inconvenience caused.

## Figures and Tables

**Figure 4.**
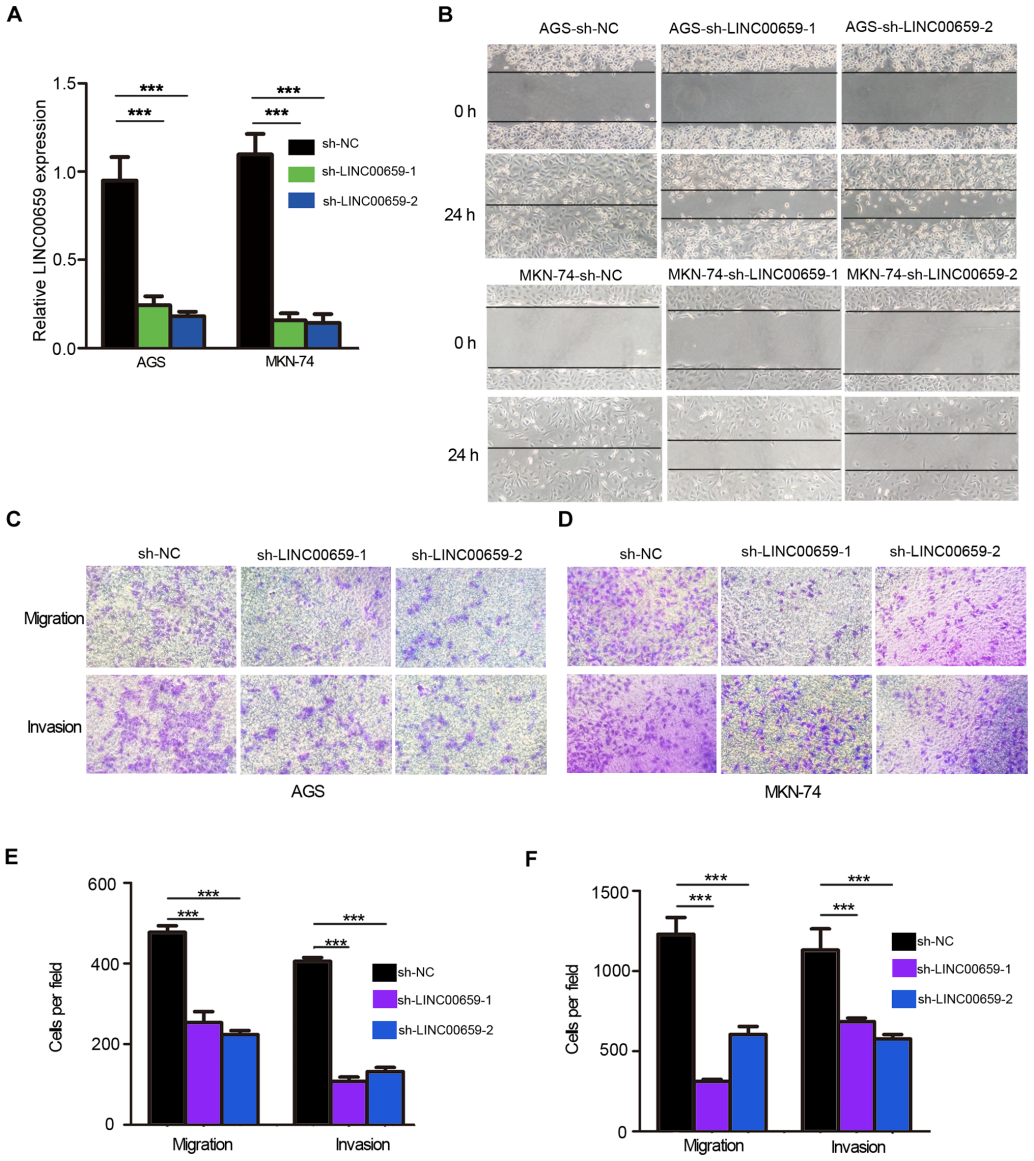
Functions of LINC00659 on GC cell migration and invasion. (A) Efficiency of sh-LINC00659, which was used to knock down LINC00659 expression, was detected in AGS and MKN-74 cells. (B) Wound healing assay revealing the migratory abilities of AGS and MKN-74 cells after knocking down LINC00659 with sh-LINC00659 (magnification, ×200). After knocking down LINC00659 expression with sh-LINC00659, cell migration and invasion assays were performed in (C) AGS and (D) MKN-74 cells (magnification, ×200). Bar charts show the quantification of the data in (E) AGS and (F) MKN-74 cells, showing the mean number of migrating/invading cells (± SD) per microscopic field from triplicate samples. Statistical analysis was performed using ANOVA with Fisher's LSD post-hoc test. ***P<0.001. sh-NC, short hairpin RNA negative control.

